# Comment on “Minilaparotomy Hysterectomy as a Suitable Choice of Hysterectomy for Large Myoma Uteri: Literature Review”

**DOI:** 10.1155/2016/7476150

**Published:** 2016-10-11

**Authors:** Flavia Sorbi, Francesco Magni, Massimiliano Fambrini

**Affiliations:** ^1^Department of Biomedical, Clinical and Experimental Sciences, Division of Obstetrics and Gynecology, University of Florence, Florence, Italy; ^2^University of Florence, Florence, Italy

We read with interest the article by Sato and Fukushima titled “Minilaparotomy Hysterectomy as a Suitable Choice of Hysterectomy for Large Myoma Uteri: Literature Review” [1]. Although this article does an excellent job in discussing benefit of the minilaparotomy approach to perform hysterectomy when laparoscopic or vaginal approach is hampered by the weight of the uterus, the study demonstrates that minilaparotomy should be considered as part of the minimally invasive armamentarium of surgical approaches for hysterectomy offered to patients with myomas.

However, further reflections can be made on minimally invasive surgery and myomas. Indeed, myomas occur mainly in women aged younger than forty, the majority of whom wish to preserve their fertility. Therefore, minilaparotomy should be considered for myomectomy as well as for hysterectomy.

We reported a large series of consecutive myomectomies performed through minilaparotomy [2]. Our study showed that more than 85% of women requiring myomectomy could be successfully managed through a 4–8 cm transverse skin incision. Failure rate was low (5.3%) and mainly associated with BMI ≥30. The mean operative time was 57 min and no wound infections or dehiscences occurred. The median hospital stay was 2.5 days. Interestingly, minilaparotomy was feasible among 95% of patients who had had previous surgery; hence this approach might be better in these patients where vaginal or laparoscopic approaches can be difficult.

Minilaparotomy offers many of the advantages of minimally invasive surgery including minimal bowel manipulation due to the exteriorization of uterine body, limited intraoperative parietal blood loss surgical pain, short operative time, and reduced incidence of wound complications. As a result, it is associated with shorter length of stay and quick return to function, but without the additional costs and complications of laparoscopic myomectomy [3].

In conclusion, we believe that nowadays the issue is not only the route of hysterectomy for benign conditions but also the route of myomectomy. Indeed, myoma is the benign condition most frequently associated with contraindication to vaginal surgery, because of the weight of myomas and laparoscopic surgery and because of the risk associated with intracorporeal morcellation and with prolonged pneumoperitoneum. Consequently, we believe that minilaparotomy is an important surgical approach to keep in mind for conservative treatment of myomas.
